# Engineering of Solar Energy Harvesting Tb^3+^-Ion-Doped CdS Quantum Dot Glasses for Photodissociation of Hydrogen Sulfide

**DOI:** 10.1021/acsaem.3c01488

**Published:** 2023-08-29

**Authors:** Mohanad Al-Murish, Vijay Autade, Eric Kumi-Barimah, Rajendra Panmand, Bharat Kale, Animesh Jha

**Affiliations:** † School of Chemical and Process Engineering, University of Leeds, Leeds LS2 9JT, U.K.; ‡ Centre for Materials for Electronics Technology (C-MET), Ministry of Electronics and Information Technology (MeitY), Off Pashan Road, Panchawati, Pune 411008, India

**Keywords:** photocatalysis, hydrogen production, Q-dot glass, photodissociation, hydrogen sulfide, rare-earth doping

## Abstract

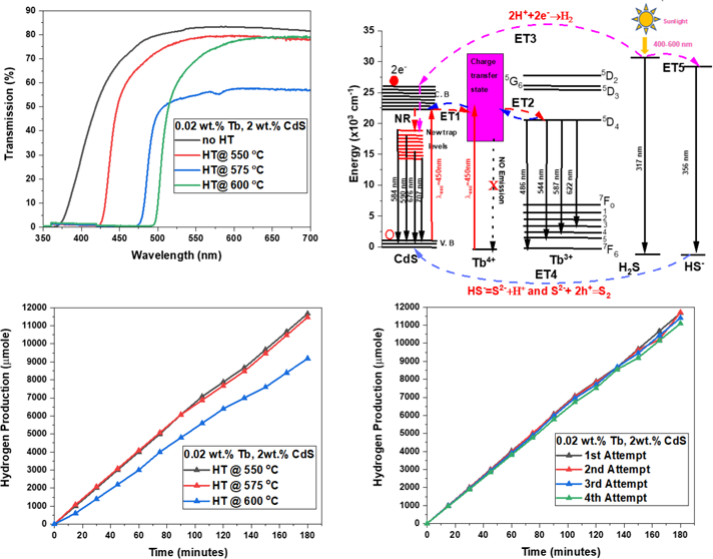

The photocatalytic properties of CdS quantum dots (Q-dots) and Tb^3+^-doped CdS Q-dots dispersed in a borosilicate glass matrix were investigated for the photodissociation of hydrogen sulfide (H_2_S) into hydrogen (H_2_) gas and elemental sulfur (S). The Q-dot-containing glass samples were fabricated using the conventional melt-quench method and isothermal annealing between 550 and 600 °C for 6 h for controlling the growth of CdS and Tb^3+^-ion-doped CdS Q-dots. The structure, electronic band gap, and spectroscopic properties of the Q-dots formed in the glass matrix after annealing were analyzed using Raman and UV–visible spectroscopies, X-ray powder diffraction, and transmission electron microscopy. With increasing annealing temperature, the average size range of the Q-dots increased, corresponding to the decrease of electronic band gap from 3.32 to 2.24 eV. For developing the model for photocatalytic energy exchange, the excited state lifetime and photoluminescence emission were investigated by exciting the CdS and Tb^3+^-doped CdS quantum states with a 450 nm source. The results from the photoluminescence and lifetime demonstrated that the Tb^3+^-CdS photodissociation energy exchange is more efficient from the excited Q-dot states compared to the CdS Q-dot glasses. Under natural sunlight, the hydrogen production experiment was conducted, and an increase of 26.2% in hydrogen evolution rate was observed from 0.02 wt % Tb^3+^-doped CdS (3867 μmol/h/0.5 g) heat-treated at 550 °C when compared to CdS Q-dot glass with a similar heat treatment temperature (3064 μmol/h/0.5 g). Furthermore, the photodegradation stability of 0.02 wt % Tb^3+^-CdS was analyzed by reusing the catalyst glass powders four times with little evidence of degradation.

## 1 Introduction

The global demand for energy consumption has been increasing steadily since the 1980s from around 87933 TWhr to around 176 431 TWh in 2021, which is attributed to the increased anthropogenic activities.^1^ The present usage of fossil fuels as an energy source has led to multiple problems, such as rising greenhouse gas emissions.^2^ Moreover, fossil fuel sources are limited and concentrated in a small region of the world and are considered unsustainable energy sources.^3^ Hydrogen sulfide (H_2_S) is a toxic gas produced in large quantities from various sources, including volcanic activity, bacterial breakdown of organic matter, and industrial processes such as natural gas processing and oil refining.^4^ The global release of H_2_S from saline marshes is estimated to be around 8.3 × 10^5^ tonnes annually. Moreover, it is also estimated that H_2_S forms around 42% of natural gas deposits.^5^ H_2_S is not classified as a greenhouse gas; however, it is an atmospheric pollutant and poisonous, even below 50 ppm level on prolonged exposure may lead to the damage of respiratory organs.^6^ In the presence of sunlight, the hydrogen sulfide in the atmosphere may photodissociate and the sulfur and hydrogen may oxidize to form greenhouse gases, namely, SO_2_ and H_2_O. Regions lacking fossil fuels have to import the oil/gas using sea transport, which also contributes to the overall greenhouse gas (GHG) emissions. Therefore, alternative green energy sources are needed to support the anthropogenic demand. In this respect, a sustainable methodology for hydrogen production may be able to offer a solution to displace the fossil fuel-based economy.^7−9^ The emission from hydrogen combustion is water vapor which is a highly potent GHG; however, the atmospheric water cycle historically has managed and controlled the overall moisture in the terrestrial atmosphere. Currently, hydrogen is produced by various methods such as natural gas steam reformation, coal and biomass gasification, thermo-chemical, nuclear, and water electrolysis, photocatalytic and photo-electro-dissociation of water or H_2_S splitting, etc.^4,10^

The photocatalytic decomposition of H_2_S is a cost-effective and eco-friendly approach for hydrogen gas production for future energy demands. The water-splitting reaction requires 285.83 kJ/mol; by comparison, the H_2_S decomposition reaction required much less energy, i.e., 79.9 kJ/mol for producing the same volume of H_2_ gas.^11^ Growing concerns about the environment and resource utilization have pushed the research for novel methodologies to convert H_2_S into S and H_2_ as green fuel using solar energy.^12,13^ Currently, researchers have focused on developing photocatalysts and cocatalysts capable of reducing the dissociation energy of H_2_S to increase H_2_ yield using UV-light sources, i.e., 300–450 nm.^14^

It is possible to utilize the abundant solar radiation for photocatalytic reactions to convert toxic pollutants such as H_2_S to clean energy (H_2_).^7^ Searching for appropriate photocatalysts is still challenging because photocatalytic efficiency is limited due to the band gap distribution of electronic states. Consequently, most photocatalysts work only by absorbing a limited part of the overall solar radiation, and any carrier states generated thereof could suffer from fast recombination.^15^ As a result, the energy transfer for molecular dissociation is adversely affected by the lack of efficient carrier availability during the excitation process.

Over the past years, various semiconductors, such as TiO_2_ and Bi_2_S_3_, have been explored and used as effective catalysts for photocatalytic H_2_S splitting.^16−19^

**Figure 1:**
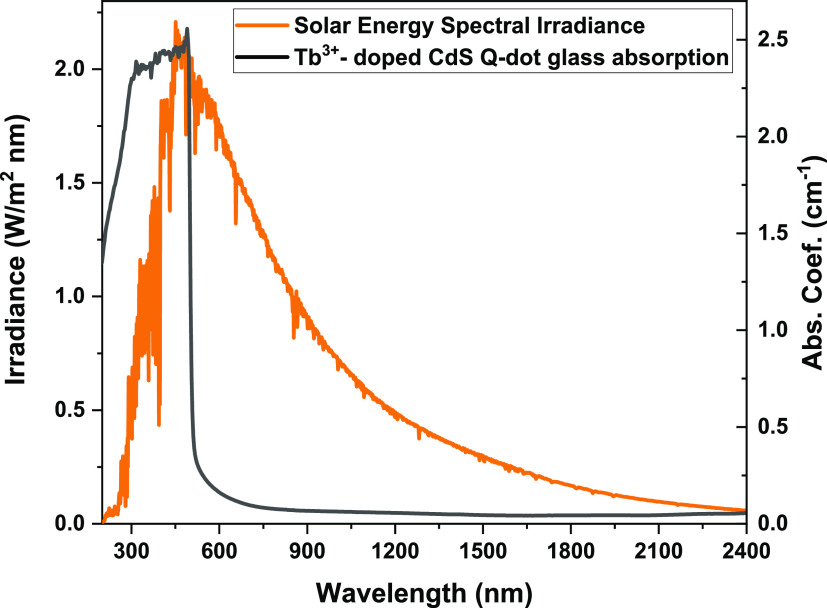
Comparison of the solar radiation spectrum—replotted using data from Gueymard—with the absorptivity of Tb^3+^-CdS Q-dot glass. Adapted with permission from ref (21). Copyright 2004 Elsevier.

Amongst the group of semiconductor photocatalysts, cadmium sulfide (CdS) has gained significant attention for hydrogen evolution owing to its outstanding physical and chemical properties at the nanoscale level.^20^ Particularly, the wide band gap energy of bulk crystalline CdS (*E*_g_ ∼ 2.42 eV) makes it highly suitable for applications in solar energy harvesting as it absorbs light around 514 nm which is the peak of visible radiation in the terrestrial solar spectrum. An example of the absorption of Q-dot glass is shown in Figure 1, and it demonstrates the overlap with terrestrial solar radiation. In this study, we have designed a process which allowed us to tailor the absorption spectrum of Q-dot glasses.

Notably, because of the above characteristics, CdS is considered one of the most desirable semiconductor Q-dot materials for H_2_ generation by splitting water or H_2_S. However, using CdS as a photocatalyst is limited due to its photo-oxidation properties, fast electron–hole recombination, and few catalytic sites owed to nanosized structure agglomeration.^22,23^ On the other hand, various strategies have been developed to overcome these limitations and enhance the photocatalytic properties of CdS. For instance, Shi et al.^23^ fabricated N-doped carbon dots/cadmium sulfide composites (N-CDs)/CdS via the hydrothermal method with good dispersion and homogeneity for photocatalytic hydrogen production. This composite of (N-CDs)/CdS exhibited high catalytic activity and stability for hydrogen production, which may be attributed to strong visible and infrared light absorption. Furthermore, Guo et al.^24^ synthesized 0D/2D CdS/α-Fe_2_O_3_ Z-Scheme heterojunction photocatalysts using a solvothermal method for photocatalytic splitting of water into hydrogen production. A high rate of photocatalytic hydrogen evolution was obtained ascribing to a larger surface area, more active sites, and reduced recombination of electron–hole pair in CdS/α-Fe_2_O_3_.

In this article, we aimed to overcome the abovementioned limitations by chemically dispersing the CdS Q-dots in a borosilicate glass matrix and codoping CdS with a Tb^3+^ ion in the form of terbium oxides (Tb_2_O_3_). Dispersion of CdS Q-dots in a borosilicate glass matrix not only reduces the risk of photo-oxidation but also ensures that they are safely contained within the corrosion- and heat-resistant borosilicate glass matrix.^25^ Despite innumerable articles on the properties of CdS Q-dots in inorganic glasses, the data on the engineering applications has been limited.^26,27^ For doping CdS, the tellurite (TeO_2_), germanate (GeO_2_), and borosilicate glasses were used as host materials for making Q-dot-doped glass.^28^ It was reported that the crystallization of semiconductor nanoparticles in the glass matrix is dependent on the chemistry of the host glass, which then controls the crystallization dynamics for the growth of Q-dots.^29^ Recently, dimetal chalcogenides, namely, the nanoscale particles of Cd(S, Se), MoS_2_, and Bi_2_S_3_ Q-dot particles have been studied for the photodissociation of H_2_S for hydrogen generation.^11,18^ As shown in Table 1, the literature reveals that the rate of hydrogen production from H_2_S is influenced by several factors, including the wavelength and power density from the source of illumination, the type of solution medium in the photoreactor, the average particle size of the catalyst, the amount of catalyst used in the medium, and the size of the quantum dots. The high evolution rates observed in Table 1 for germanate glasses dispersed with bismuth sulfide are attributed to two primary factors. First, the ultraviolet edge of germanate glasses is resonant with the UV source in the Xe lamp, leading to increased excitation of the electronic states in the GeO_2_ glass matrix. Second, the bismuth sulfide exhibits strong absorption in the UV–vis region,^30^ indicating a large probability of excited state transition and energy exchange between the GeO_2_ and Bi_2_S_3_, which increases the photodissociation efficiency. In the present work, our focus is on the use of borosilicate glass, which is widely used as a heat-resistant material, and the reactors may be fabricated at a much lower cost than a germanium oxide-based glass. GeO_2_ is a rare semiconductor oxide and hard to extract from natural minerals.

**Table 1:** Summary of Literature on Hydrogen Production through Photodissociation with Different Factors, Using Various Quantum Dot and Quantum Dot Glass Catalysts

type of catalyst	media in the photoreactor	mass of used catalyst (g)	quantum dot size (nm)	particle size of catalyst (nm)	light source	hydrogen evolution rate (μmol/h)	reference
CdS bulk particle	H_2_S + (0.5 M) KOH	0.5		1000	450 W Xe lamp with a cutoff filter (>420 nm)	1010	(31)
CdS nanoparticle	H_2_S + (0.5 M) KOH	0.5	5–7	5–7	450 W Xe lamp with a cutoff filter (>420 nm)	2945	(31)
0.5 wt % CdS in borosilicate glass	H_2_S + (0.5 M) KOH	1	2.5	2000–5000	450 W Xe lamp with a cutoff filter (>420 nm)	3570	(25)
0.5 wt % CdS in borosilicate glass	H_2_S + H_2_O	1	2.5	2000–5000	450 W Xe lamp with a cutoff filter (>420 nm)	2320	(25)
2 wt % CdS in germanate glass heat-treated at 450 °C for 12 h	H_2_S + (0.5 M) KOH	0.5	4–5	not available	450 W Xe lamp with a cutoff filter (>420 nm)	3780	(28)
2 wt % CdS_0.5_Se_0.5_ in germanate glass heat-treated at 425 °C for 8 h	H_2_S + (0.5 M) KOH	1	4–5	not available	450 W Xe lamp with a cutoff filter (>420 nm)	8164.53	(32)
0.3 wt % Bi_2_S_3_ in germanate glass heat-treated at 450 °C for 8 h	H_2_S + (0.5 M) KOH	1	1–2	not available	450 W Xe lamp with a cutoff filter (>420 nm)	11 541.22	(30)
0.3 wt % Bi_2_S_3_ in germanate glass heat-treated at 450 °C for 8 h	(0.25 M) Na_2_S + (0.35 M) Na_2_ SO_3_	1	1–2	not available	450 W Xe lamp with a cutoff filter (>420 nm)	25.6	(30)
0.7 wt % Bi_2_S_3_ in borosilicate glass heat-treated at 550 °C for 8 h	H_2_S + (0.5 M) KOH	1	4.6	not available	300 W Xe lamp	6418.8	(19)

Since bare Q-dots of chalcogenide semiconductors are susceptible to photodegradation, our goal in this investigation is to disperse the Q-dot glass in a thermal-resistant borosilicate matrix and also enhance the solar radiation capture capacity of glass for photocatalytic dissociation of H_2_S by controlling the Q-dot particle size during the heat treatment process of quenched glass. Also, at present, there is no literature showing any data on the effect of rare-earth ions doped with CdS in a borosilicate glass matrix. The reason for incorporating a rare-earth ion (e.g., Tb^3+^) in CdS is relevant because the RE ions have strong absorption bands in the UV–visible region due to the unique (4f)^n^5d^y^ or (4f)^n^6s^w^ structures. Our hypothesis is also based on the fact that the presence of RE ions in CdS may also enhance the crystallization tendency due to the differences in the ion-coordination environment (Tb^3+^ is octahedrally coordinated, Cd^2+^ is tetrahedrally coordinated in an sp^3^ configuration). Our final hypothesis is that none of the photocatalytic models reports the energy exchange mechanism, and in this context, the comparison of the photocatalytic effect in CdS-doped Q-dot glass and Tb^3+^-CdS-doped Q-dot glasses may be possible to analyze in detail for improving the hydrogen production. There is also a limited investigation on RE-ion-doped Cd(S, Se, Te) systems for photodegradation stability, which is important for engineering applications. Hamnabard et al.^33^ showed that the doping of a Yb^3+^ ion at 0.1 mol % in CdTe improved the stability against photodegradation. For photocatalytic applications of CdS and Tb^3+^-CdS Q-dots doped in a glass matrix, we have investigated a number of standard materials’ structural and spectroscopic analysis techniques for demonstrating and validating the energy exchange model.

## 2 Experimental Section

### 2.1 Material Synthesis

In this study, a borosilicate glass composition consisting of 46 wt % SiO_2_, 6 wt % B_2_O_3_, 12 wt % ZnO, 17 wt % K_2_O, 9 wt % Na_2_O, 4 wt % MgO, 5 wt % TiO_2_, and 1 wt % starch was adopted. 2 wt % CdS was added to the glass composition in powder form, and Tb^3+^ ions in the form of Tb_4_O_7_ powder were codoped with the CdS glass mixture as 1 and 2 wt % concentrations with respect to the amount of the CdS used. The exact glass composition with dopant concentrations is shown in Table 2.

**Table 2:** Quantum Dot Glass Sample Composition with Dopant Concentration

sample	SiO_2_	B_2_O_3_	ZnO	K_2_O	Na_2_O	MgO	TiO_2_	starch	CdS	Tb_4_O_7_
host glass	46.39%	6.20%	12.09%	16.79%	9.20%	3.59%	4.74%	0.99%	0.00%	0.00%
2 wt % CdS only	45.47%	6.08%	11.84%	16.45%	9.02%	3.52%	4.65%	0.97%	2.00%	0.00%
1 wt % Tb in CdS i.e., (0.02 wt % Tb in 2 wt % CdS)	45.46%	6.08%	11.84%	16.45%	9.02%	3.52%	4.65%	0.97%	2.00%	0.02%
2 wt % Tb in CdS i.e., (0.04 wt % Tb in 2 wt % CdS)	45.45%	6.08%	11.84%	16.44%	9.02%	3.52%	4.65%	0.97%	2.00%	0.04%

It is worth noting that in this glass composition, B_2_O_3_, ZnO, and TiO_2_ demonstrate a tendency for phase separation in silicate glass which is well documented in the glass-ceramic literature.^34^ Addition of chalcogenides, namely, CdS, further enhances the intrinsic tendency for phase separation and, therefore, promotes crystallization upon heat treatment. Furthermore, rare-earth ions such as terbium are known to have sub 1 mol % solubility in silicate glass and tend to segregate on the sub-nanometer scale in the silicate matrix in the absence of alumina and phosphate.^35^

Raw materials used to prepare the glass samples were made of analytical grade with purity <99%, which were purchased from S.D. Fine Chemicals Ltd. (India). All glass compositions with the dopants, described above, were weighed and mixed thoroughly using a mortar and a pestle to obtain a homogeneous mixture. The powdered mixture after grinding and mixing was transferred into an alumina crucible for melting using an electrically heated muffle furnace (Lenten EHF 1700) at 1250 °C for 3 h in air. After that, the molten glass was poured into a preheated brass mold at 550 °C for quenching in air. Each glass sample was cut for characterization. The glass transition temperature (*T*_g_) and glass crystallization temperature (*T*_c_) of 3 wt % CdS in a borosilicate glass was reported to be 534 and 615 °C, respectively.^36^ Therefore, glass samples were isothermally heat-treated for 6 h between 550 and 600 °C to control the growth of CdS Q-dots in the glass matrix. After heat treatment, each glass sample was polished for structural and spectroscopic characterizations. For photocatalytic studies of H_2_S dissociation, part of the polished glasses was cut and pulverized into a fine powder with a mean size range from 28 to 30 μm for photocatalytic studies. Particle size distribution data could be found in Figure S1.

### 2.2 Structural and Spectroscopic Characterization

The structural information of the as-prepared 2 wt % CdS-doped borosilicate glass and samples heat-treated from 550 to 600 °C were initially studied by employing a Cu Kα radiation Bruker D8 Advance X-ray diffractometer (XRD) at 40 mA and 40 kV with a 2θ range of 15–60° in a step size of 0.008 for about 14.5 h. Furthermore, the XRD measurement was repeated for 0.02 and 0.04 wt % Tb^3+^-CdS-doped borosilicate glass samples heat-treated at 600 °C.

Raman spectroscopy measurements for as-prepared and heat-treated at 550 and 600 °C CdS-doped borosilicate glass samples were collected using a Renishaw inVia Raman microscope with a 514 nm laser in the spectral range of 150–1000 cm^–1^. A similar measurement was conducted for 0.02 and 0.04 wt % Tb^3+^-CdS-doped borosilicate glasses heat-treated at 600 °C.

The microstructural properties of the Q-dot glass samples heat-treated at 550 °C were investigated by using transmission electron microscopy (TEM-JEM-2200). Additionally, a PerkinElmer Lambda 950 spectrometer was used to measure transmittance for as-prepared and heat-treated Q-dot glass samples at room temperature in a wavelength range of 350–700 nm. The photoluminescence (PL) emission was measured at room temperature by adopting an FLS 980 spectrometer (Edinburgh Instruments, U.K.) with a 450 nm pigtailed laser diode (Thorlabs, U.K.). The 450 nm continuous laser beam was mechanically chopped using an optical chopper rotating disk consisting of regularly spaced wide slits to measure PL decay curves. The continuous laser beam passes through the rotating disk providing an on-and-off switch of the laser beam to generate an optical pulse for the decay curve measurements. The PL decay curve was fitted using a single exponential function via Origin Pro, and the average lifetime was estimated.

### 2.3 H_2_S Formation and Photocatalytic Study

The hydrogen production through photocatalytic reactions was conducted in ambient conditions and under direct natural sunlight on sunny days between March and May from 10 am to 3 pm in Pune, India (location 18.52°N 73.86°E). The global solar light intensity was measured using a MEXTECH digital Lux meter (Model LX1010B) with incident solar light intensity ranging from 97000 (808 W/m^2^) to 99000 Lux (825 W/m^2^).

**Figure 2:**
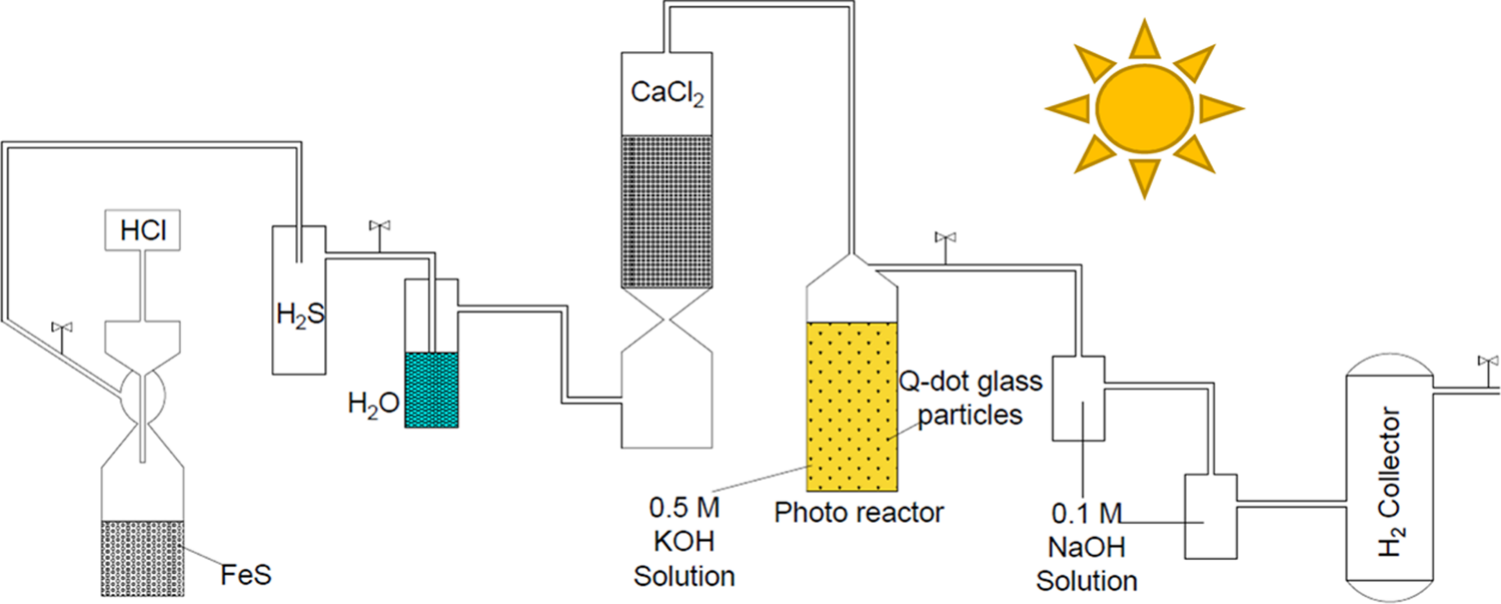
Schematic diagram showing H_2_S production and H_2_ generation using the photocatalysis process.

Figure 2 shows the experimental setup for H_2_S production and the photocatalytic reaction of H_2_S into H_2_ gas. H_2_S was produced by the reaction between FeS (S) and HCl (aq) as shown by eq 1. The produced H_2_S was passed through the calcium chloride (CaCl_2_) chamber to absorb moisture. Then, H_2_S was collected in an empty trap.
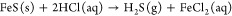
1In this experiment, 0.5 g of the photocatalyst (crushed Q-dot glass powder containing CdS or Tb^3+^-CdS) was added to the photoreactor containing 750 mL of 0.5 M KOH as seen in Figure 2. This suspension in the photoreactor was stirred at 1000 rpm and purged with argon for 1 h. After that, H_2_S was bubbled into the photoreactor at a rate of 2.5 mL/min under natural sunlight. Subsequently, the generated H_2_ gas was collected using a graduated glass burette and analyzed by gas chromatography (Model Shimadzu GC-14B, MS-5Å column, TCD, Ar carrier). Escaping H_2_S from the photoreactor was trapped in the next vessel containing 10% NaOH solution. The sulfur remains in the photoreactor as suspended solid particles in the solution.

## 3 Results and Discussion

### 3.1 X-ray Diffraction (XRD)

**Figure 3:**
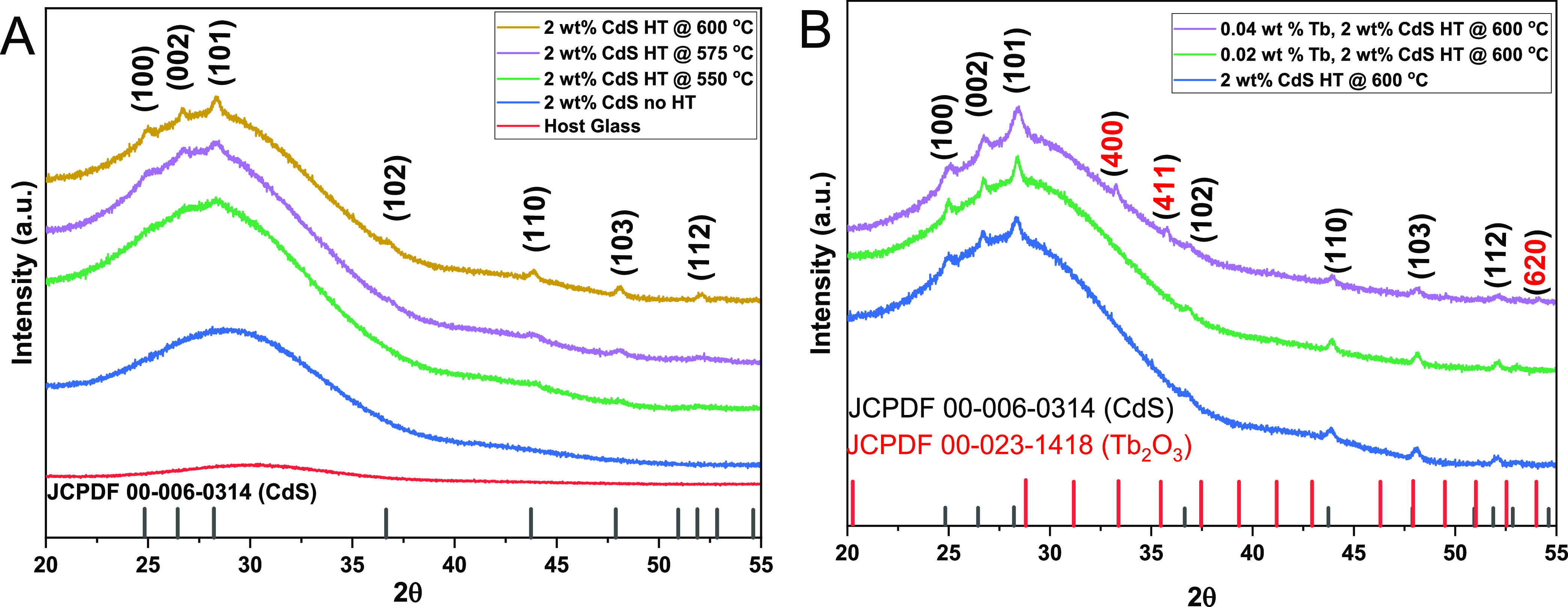
XRD pattern of (A) host glass and 2 wt % CdS glasses at different heat treatment temperatures. (B) 2 wt % CdS and 0.02 and 0.04 wt % Tb^3+^-CdS heat-treated at 600 °C for 6 h.

The X-ray diffraction patterns of 2 wt % CdS and 0.02 and 0.04 wt % Tb-doped CdS Q-dot glasses at different heat treatment temperatures are shown in Figure 3. The broadband in the 2θ range of 22–40° presents evidence of an amorphous silicate glass matrix structure in all samples. Figure 3A shows the XRD patterns for the borosilicate host glass, as-prepared 2 wt % CdS sample, and 2 wt % CdS heat-treated at 550, 575, and 600 °C. The results in Figure 3A showed that as the heat treatment temperature is increased, the crystalline peaks emerge on the background of the amorphous borosilicate host glass. According to HighScore analysis, the diffraction patterns of 2 wt % CdS heat-treated at 550, 575, and 600 °C exhibit CdS peaks at 24.83, 26.65, 28.38, 36.65, 43.74, 47.9, and 51.9° corresponding to (100), (002), (101), (102), (110), (103), and (112) planes, respectively. These peaks match the hexagonal crystal structure of CdS (JCPDF reference code 00-006-0314).

Figure 3B illustrates a comparison of the XRD patterns for 2 wt % CdS and 0.02 and 0.04 wt % Tb^3+^-doped CdS Q-dot glass samples heat-treated at 600 °C. The patterns show that the addition of Tb^3+^ ions enhanced the intensity of the crystalline peaks. The hexagonal CdS crystalline peaks observed for the 2 wt % CdS were also seen for samples containing 0.02 and 0.04 wt % Tb^3+^ ions heat-treated at 600 °C as depicted in Figure 3B. However, for the sample doped with 0.04 wt % Tb^3+^ ions, three additional XRD peaks emerged at 2θ of 33.29, 35.82, and 54.11°. These peaks correspond to the (400), (411), and (620) phases of the cubic crystal structure of Tb_2_O_3_ (JCPDF reference code 00-023-1418). This observation confirms that Tb^3+^ ions have been successfully incorporated into the Q-dot glass matrix. The presence of crystalline peaks on the samples heat-treated between 550 and 600 °C confirms nanocrystalline growth in the glass matrix.

### 3.2 Raman Spectroscopy

**Figure 4:**
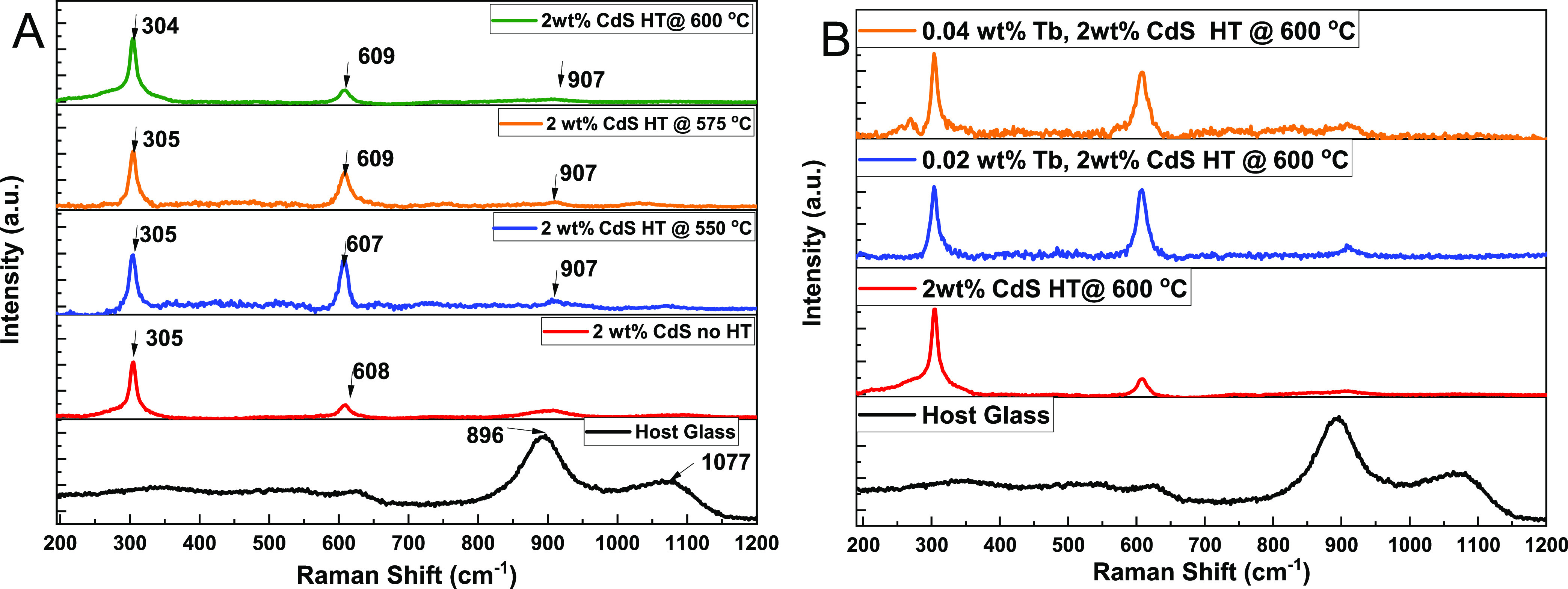
Raman spectra of (A) 2 wt % CdS glass at different heat treatment temperatures. (B) 2 wt % CdS, 0.02 wt % Tb doped in 2 wt % CdS, and 0.04 wt % Tb doped in 2 wt % CdS heat-treated at 600 °C for 6 h.

Figure 4A presents the Raman spectra of the host glass and as-prepared 2 wt % CdS glass sample with heat treatment at 550, 575, and 600 °C. The host glass reveals a weak Raman band at 627 cm^–1^ and two intense broad Raman bands at 896 and 1077 cm^–1^ corresponding to ring-type metaborate groups and SiO_4_ tetrahedra with two nonbridging and one nonbridge oxygen ions.^37^ The Raman spectra of the as-prepared 2 wt % CdS glass and heat treatment at 550, 575, and 600 °C samples show two main Raman vibration peaks, which are centered at ∼305 and ∼609 cm^–1^. The peaks centered at 305 and 609 cm^–1^ are associated with the longitudinal optical (LO) phonon modes of CdS, namely, the fundamental-overtone (1LO) and first-overtone (2LO), respectively, as reported by Vetchinnikov et al.^38^

Similarly, Figure 4B shows Raman spectra of the 2 wt % CdS, 0.02 wt % Tb doped in 2 wt % CdS, and 0.04 wt % Tb doped in 2 wt % CdS glasses heat-treated at 600 °C. These spectra exhibit similar Raman vibrational peaks at ∼305 and 609 cm^–1^. Notably, in Figure 4B, it was observed that the intensity and broadening of the 1LO overtone mode peak decrease with increasing doping concentration of Tb^3+^ ions, while the intensity of the 2LO overtone mode shows a slight increase.

These variations in the intensities of the 1LO and 2LO Raman vibration modes can be attributed to the phase transition caused by structural modification resulting from Tb^3+^ ion doping, leading to an electron–phonon coupling effect.^39^ According to the findings of Lin et al., the magnitude of electron–phonon coupling primarily depends on the heat treatment temperature and the size of the grown nanocrystals, irrespective of the crystal structure.^40^

This Raman data complements the XRD data and provides additional evidence of the successful in situ crystallization of CdS quantum dots inside the glass host.

### 3.3 Transmission Electron Microscopy (TEM)

**Figure 5:**
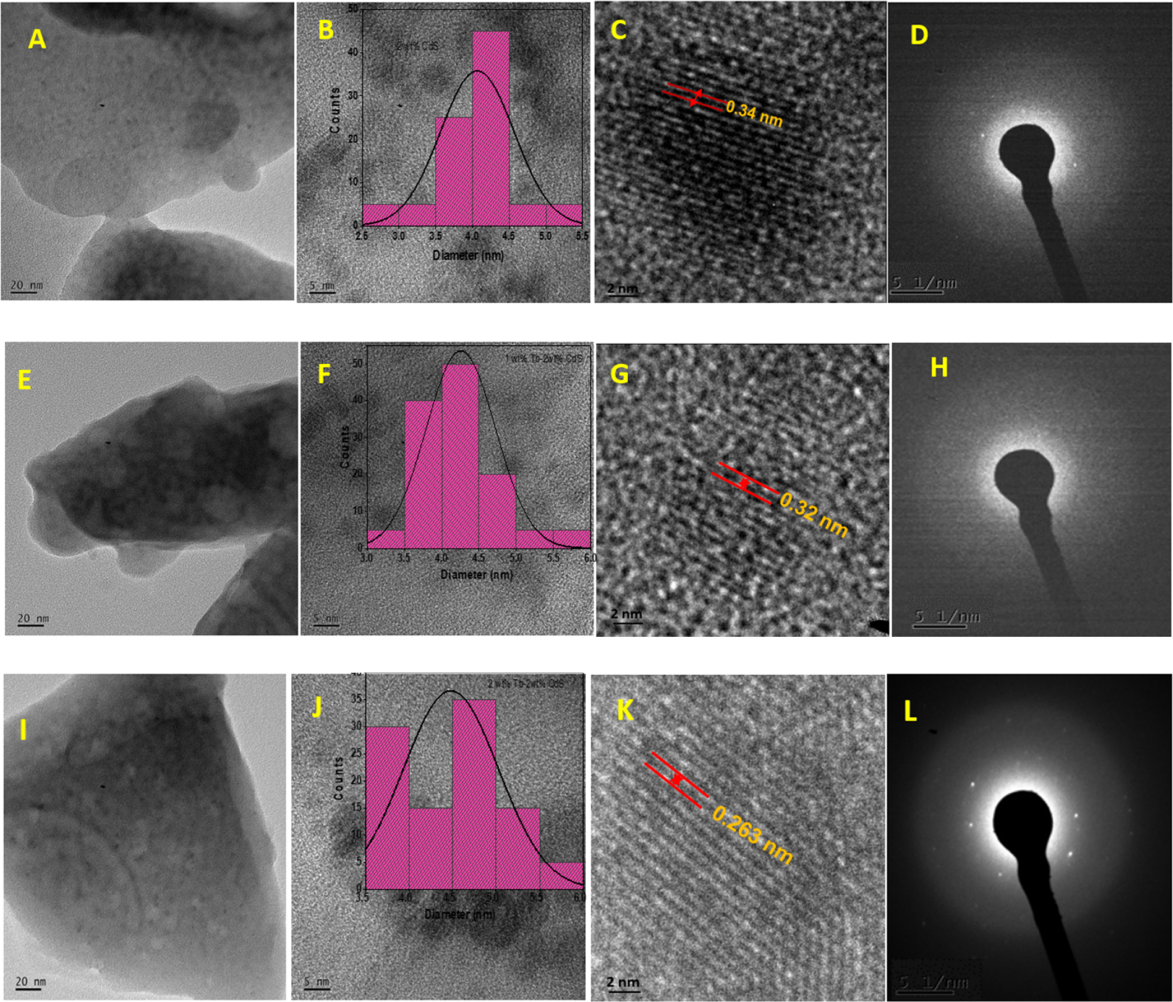
TEM, HR-TEM, and diffraction pattern images of Q-dots formed via heat treatment at 550 °C in a silicate glass matrix: (i) CdS Q-dot (A–D), (ii) 0.02 wt % Tb-doped CdS (E–H), and (iii) 0.04 wt % Tb-doped CdS (I–L).

Field emission transmission electron microscopy (FE-TEM) analysis was performed to investigate the microstructural properties of the formed Q-dots after 6 h of annealing at 550 °C. The obtained FE-TEM images in Figure 5A–L depict various aspects, including the morphological features (Figure 5A,E,I), particle size and size distribution histogram obtained from high-resolution TEM (HR-TEM) (Figure 5B,F,J), lattice fringes of the Q-dot particles (Figure 5C,G,K), and selected area electron diffraction (SAED) patterns (Figure 5D,H,L). The SAED patterns in Figure 5D,H,E confirm the polycrystalline nature of the Q-dot glass. Moreover, the results indicate that the Tb^3+^ doping of CdS Q-dots does not appear to significantly influence the morphology or the phase constitution characteristics. The FE-TEM micrographs and particle size distribution histograms in Figure 5B,F,J reveal a size range of 3–5, 3–6, and 4–6 nm for the 2 wt % doped CdS and 0.02 wt % and 0.04 wt % Tb^3+^-doped CdS quantum dots, respectively.

Analysis of the FE-TEM images suggests a growth mechanism resembling the Ostwald ripening phenomenon, resulting in an average CdS Q-dot size of 5 nm within the borosilicate glass matrix. The SAED and lattice fringe analysis demonstrate d-spacing values of 0.34, 0.32, and 0.26 nm, corresponding to the (002), (101), and (102) planes of the hexagonal JCPDF reference code 00-006-0314 polytype of CdS and Tb^3+^-doped CdS solid solution phase, respectively.

Since the ionic radii of Tb^3+^ (93.2 pm) and Cd^2+^ (97 pm) are comparable, the TEM analysis provides limited evidence at the Q-dot scale regarding the solubility of Tb^3+^ ions in the CdS Q-dot glass matrix following heat treatment above Tg. Additionally, the crystal structures of CdS and Tb_2_S_3_ are based on zinc blend/wurtzite and rock salt, respectively. The crystallographic habit planes (101) may be shared, promoting isotropic or unidirectional growth at the quantum scale, depending on the atomic packaging density of the dominating growth plane.

As discussed in our previous article,^29^ the ionic radius of the S^2–^ anion is 170 pm. This indicates that the average distance between two sulfur anions (S^2–^) would not exceed 340 pm. Considering the thermodynamic stability of CdS and Tb_2_S_3_ at the melting temperature of borosilicate glass, it is expected that these two sulfides with over 270 pm cation (Tb^3+^, Cd^2+^)–anion (S^2–^) distance might be virtually insoluble in the borosilicate glass matrix with less than 175 pm (Si^4+^–O^2–^) bond distance.

For the overall stability of borosilicate glass, the soluble species must have comparable bond lengths to be accommodated within the borosilicate matrix. The apparent disparity between the bond lengths of the borosilicate and chalcogenide crystallites is likely to force the CdS Q-dots to nucleate within the amorphous matrix, which is the driving force for promoting nucleation and growth, leading to Oswald ripening.^38,41−44^ Above *T*_g_, the thermal energy promotes ionic conductivity leading to the formation of CdS and Tb-doped CdS phases.

### 3.4 UV–Vis

**Figure 6:**
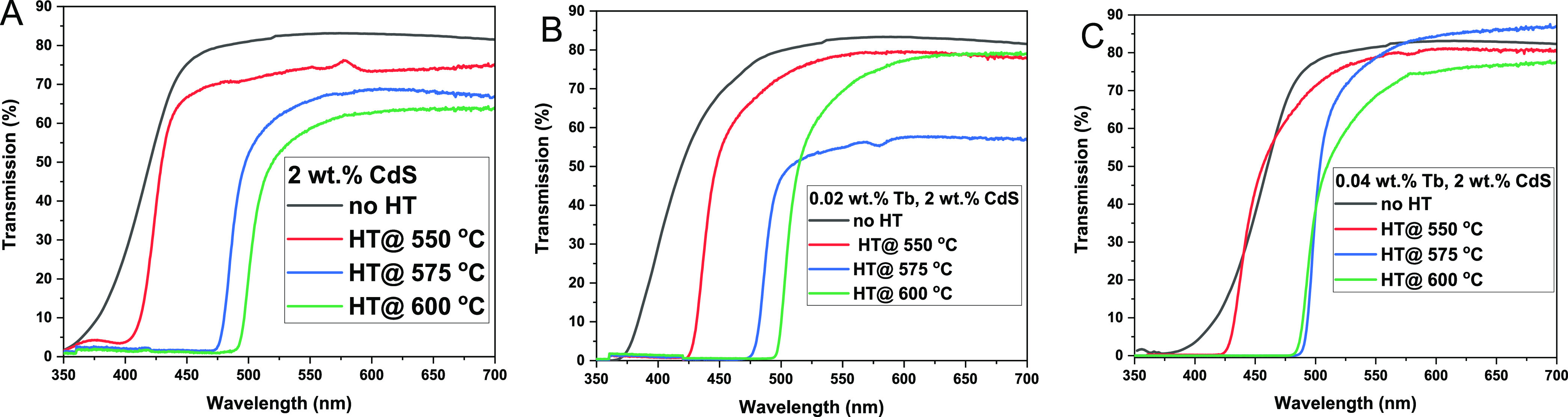
Optical transmittance spectra for (A) 2 wt % CdS, (B) 0.02 wt % Tb-doped CdS, (C) 0.04 wt % Tb-doped CdS in the borosilicate glass matrix at different heat treatment temperatures.

Figure 6A–C depicts the UV–visible optical transmission spectra for the 2 wt % CdS samples (A), 0.02 wt % Tb^3+^-CdS (B), and 0.04 wt % Tb^3+^-CdS (C) Q-dot glasses at different heat treatment temperatures. From Figure 6B,C, it is clear that the UV–visible absorption tail of as-prepared Tb^3+^-CdS Q-dots glasses extends from 375 to 410 nm as the Tb_4_O_7_ concentration increases from 0.02 to 0.04 wt %. This can be attributed to the oxidized species of Tb-doped CdS Q-dots from the occupied O-orbitals to unoccupied Tb^4+^ orbitals charge transfer in the glass matrix.^45^ The strong and broad absorption band of the Tb^4+^ charge transfer transitions appear from 350 to 600 nm as reported in the literature.^46−48^ Moreover, in Figure 6B,C, the strong absorption of the CdS Q-dots in the UV–visible spectral range suppresses the absorption bands of Tb^3+^ ions in this spectral region.

The Tauc equation was utilized to estimate the optical band gap of the Q-dot glasses based on the energy-dependent absorption coefficient, which is expressed in eq 2.^49^
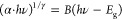
2where  is the absorption coefficient with l being the thickness of the glass sample and *T* being the UV–visible transmittance spectrum. The *ν*, *h*, and *E*_g_ are the photon’s frequency, Planck constant, optical band gap, and *B* is a constant, whereas the exponential factor, γ, depends on the electron transition responsible for the optical band gap which is known to be 1/2.

Furthermore, the average Q-dots particle size embedded in the glasses was calculated using the Q-dots optical band gap obtained above due to electron–hole spatial correlation and the Brus model as shown in eq 3.^29^
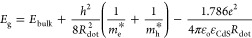
3where *E*_bulk_ is the optical band gap energy of the bulk semiconductor, *R*_dot_ represents the radius of the quantum dot, *m*_e_^*^ and *m*_h_^*^ are the effective masses of the excited electron and hole, respectively, and ε_o_ and ε_CdS_ are the dielectric constants of vacuum and CdS, respectively.

Table 3 below shows the calculated optical band gap from Tauc plots and Q-dot size from the Brus model. It was concluded from the spectroscopic studies that by increasing the heat treatment temperature from 550 to 600 °C, the Q-dot size increases resulting in the red shift of the electronic band gap edge from 3.31 to 2.55 eV for the 2 wt % CdS containing glass. The corresponding red shift in the band edge for 0.02 wt % Tb^3+^ and 0.04 wt % Tb^3+^-doped CdS Q-dots changed from 3.32 to 2.50 eV and 2.98 to 2.44 eV, respectively. As reported previously, the increase in Q-dots size is attributed to a decrease in optical band gap.^50−52^ According to Beydoun et al.,^53^ the semiconductor CdS Q-dots have the advantage of tuneable optical band gap, which decreases with increasing Q-dot particle size, and this was also found in our calculations as shown in Table 3. It is worth mentioning that the increase in CdS Q-dots size leads to a decrease in the mean free path of scattering. If the Q-dots size distribution is large and there are more Q-dots in the host glass, the mean free path becomes shorter; henceforth, more incident light can penetrate the material without being scattered away, resulting in a higher probability of light absorption.

**Table 3:** Band Gap and Calculated Q-Dot Size of 2 wt % CdS and 0.02 and 0.04 wt % Tb^3+^-CdS Q-Dot Glasses at Different Heat Treatment Temperatures and Different Tb^3+^ Ion Concentrations

sample	treatment temperature	band gap (eV)	calculated Q-dot size (nm)
2 wt % CdS only	without heat treatment	3.31 ± 0.02	3.5 ± 0.1
	550 °C held for 6 h	3.04 ± 0.02	4.4 ± 0.2
	575 °C held for 6 h	2.61 ± 0.01	5.1 ± 0.3
	600 °C held for 6 h	2.55 ± 0.01	5.4 ± 0.3
0.02 wt % Tb in 2 wt % CdS	without heat treatment	3.32 ± 0.02	3.6 ± 0.1
	550 °C held for 6 h	2.90 ± 0.02	4.8 ± 0.2
	575 °C held for 6 h	2.59 ± 0.01	5.1 ± 0.3
	600 °C held for 6 h	2.50 ± 0.01	5.8 ± 0.3
0.04 wt % Tb in 2 wt % CdS	without heat treatment	2.98 ± 0.02	4.6 ± 0.2
	550 °C held for 6 h	2.90 ± 0.02	4.9 ± 0.3
	575 °C held for 6 h	2.48 ± 0.01	5.9 ± 0.3
	600 °C held for 6 h	2.44 ± 0.01	6.2 ± 0.3

As seen in Table 3, the average Q-dot particle size calculated using the Brus equation is in good comparison with the data determined using TEM. However, the observed increase in the average Q-dot size between 550 and 600 °C is relatively slow due to the high viscosity of the glass matrix (10^6^–10^7^ Pa·s).^54^ It should be noted that the amorphous borosilicate network does not provide preferential growth directions for the nucleation of the CdS phase (with or without Tb_2_O_3_); thus, forcing the Q-dots to grow isotropically.

### 3.5 Photoluminescence and Lifetime (PL) Spectroscopy

**Figure 7:**
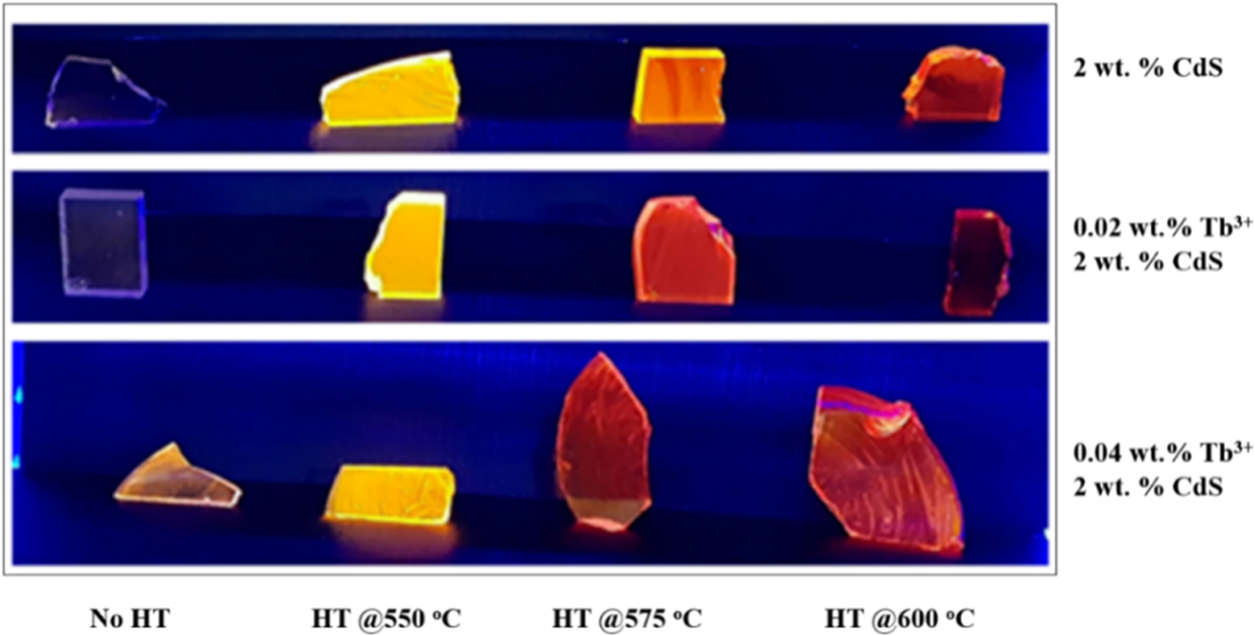
Photograph of 2 wt % CdS and 0.02 and 0.04 wt % Tb^3+^-CdS Q-dot glasses at different heat treatment temperatures under 322 nm UV light.

Figure 7 presents a visual representation of 2 wt % CdS and 0.02 and 0.04 wt % Tb^3+^-doped CdS Q-dot glasses after being exposed to a 322 nm UV light. The change in colors of the samples heat-treated between 550 and 600 °C is attributed to the increase in Q-dot size. These changes are comparable to the red shift seen in the CdS and Tb^3+^-CdS Q-dot glasses PL spectra, which are discussed below.

**Figure 8:**
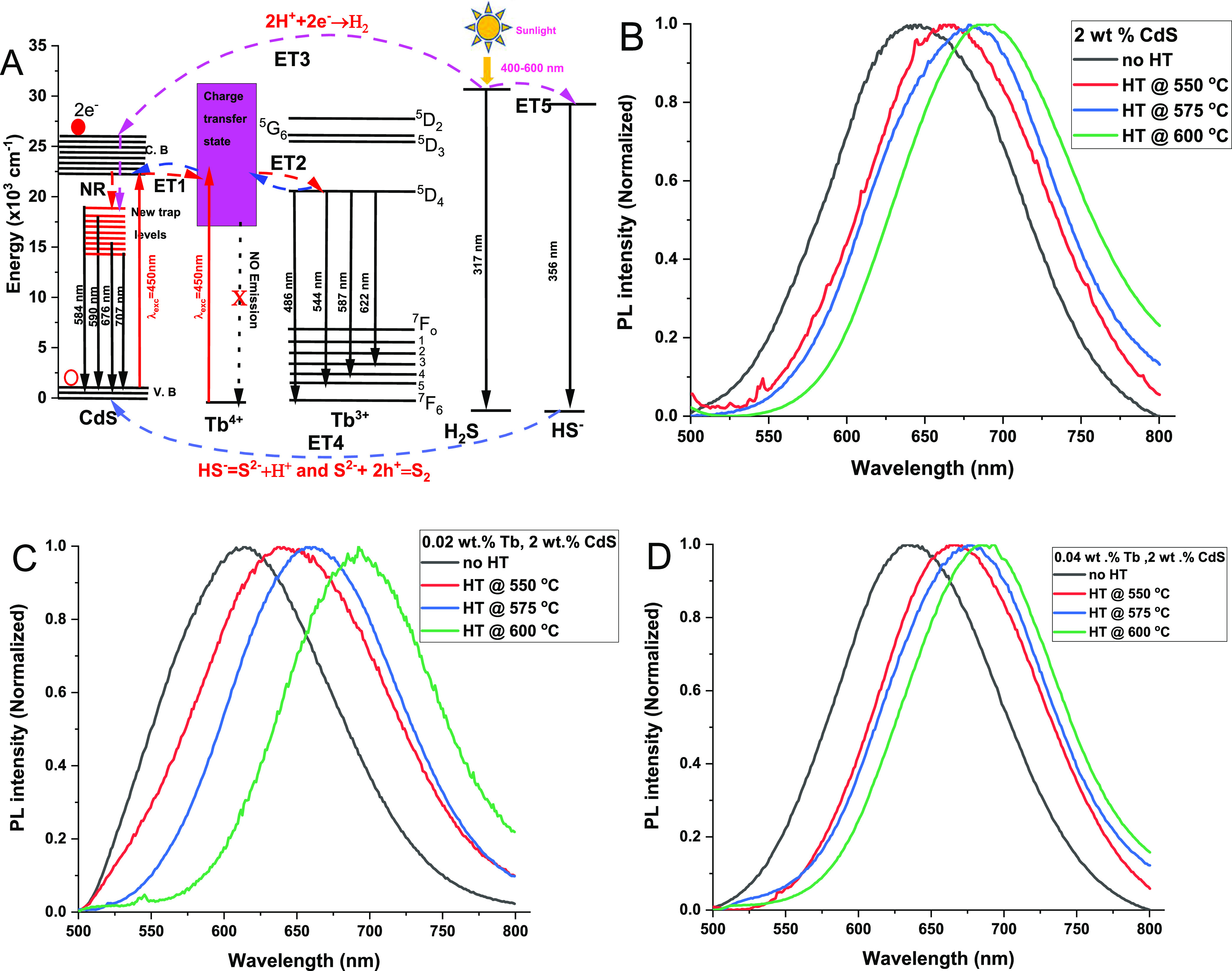
(A) Partial energy diagrams of Cd^2+^ and Tb^3+^ under 450 nm excitation showing the ground state absorption, energy transfer between the Cd^2+^ and Tb^4+^ (ET1), energy transfer between the Tb^4+^ and Tb^3+^ (ET2), energy transfer from H_2_S to Cd^2+^ (ET3), energy transfer from the ground state of the HS^–^ to the ground state of the CdS (ET4), photodissociation and energy transfer from H_2_S into HS^–^ by sunlight (ET5). 450 nm excitation photoluminescence spectra of (B) 2 wt % CdS quantum dots in borosilicate-based glass at different heat treatments. (C) 0.02 wt % Tb^3+^-CdS quantum dots in borosilicate-based glass at different heat treatments. (D) 0.04 wt % Tb-CdS quantum dot in silicate-based glass at different heat treatments.

The PL spectra analysis was performed to investigate the charge-carrier trapping, migration and transfer, and electron–hole pair recombination processes in the CdS and Tb^3+^-CdS Q-dot glasses. Room temperature PL spectra of 2 wt % CdS and 0.02 and 0.04 wt % Tb^3+^-CdS Q-dot in the borosilicate glass matrix was monitored using 450 nm laser as shown in Figure 8B–D. The 450 nm laser excites electrons from the valance band (V.B., ground state) to the conduction band (C.B., excited state) of the semiconductor CdS or from the ground state to the charge transfer state of the Tb^4+^ ion. The excited electrons undergo nonradiative decay (NR) from the C.B. to the new trap states and then emit photoluminescence through radiative decay to the V.B., as illustrated by the partial energy diagram in Figure 8A. Alternatively, the electron transfer mechanism from C.B. of CdS to the Tb^4+^ ion and vice versa may occur via the ET1 route without emitting photoluminescence emission from the Tb^4+^ion. The electron transfers from the CdS C.B. to the Tb^4+^ charge transfer state result in effective charge separation, leaving holes in the V.B. on CdS. This is because the Tb^4+^ ion has a broad absorption band in the charge transfer state located at the 300–600 nm spectral region because of its 4f^*n*–1^ to 4f^*n*–2^ transitions. Afterward, electrons can be transferred from the Tb^4+^ ion charge transfer state to the ^5^D_4_ level of the Tb^3+^ion through the ET2 route.

The PL spectra of these samples exhibit broadband in the visible to near-infrared (NIR) range from 500 to 800 nm. Upon excitation, the peak wavelength of the PL shifts toward the NIR region as the heat treatment temperature increases from 550 to 600 °C. For example, the PL peaks for the as-prepared 2 wt % CdS Q-dot glass and samples heat-treated at 550, 575, and 600 °C were found to be centered at 644, 668, 679, and 690 nm, respectively, as shown in Figure 8B. The red shift of the PL peak was ascribed to an increase in average Q-dot crystal size and newly formed recombination centers (new states) within the CdS Q-dots in the glass matrix as the heat treatment temperature increases. A similar red shift was observed for the as-prepared 0.02 and 0.04 wt % Tb^3+^-doped CdS Q-dots in the glass matrix as the heat treatment temperature increased from 550 to 600 °C as seen in Figure 8C,D. Nevertheless, the Tb^3+^ ion emission peaks centered at 488, 543, 586, and 621 nm were not observed,^55^ which is likely because the strong CdS Q-dots PL emission band overshadowed the Tb^3+^ ion transition peaks, as illustrated in Figure 8A.

The full width at half-maximum (FWHM) values were obtained by fitting the PL spectra with a Gaussian curve via Origin Pro software. For instance, the FWHM for the as-prepared 0.04 wt % Tb^3+^-CdS Q-dot glass and samples heat-treated at 550, 575, and 600 °C were found to be 130, 129, 128, and 125 nm, respectively. The changes in the FWHM with the heat treatment temperature may be attributed to the inhomogeneous broadening due to the population of the electronic states on a range of different Q-dot sizes, nucleating and growing simultaneously. Moreover, the changes in FWHM may also be attributed to the changes in the electron–phonon coupling mechanisms as the Q-dot size changes in the glass matrix.^56^ The trends of the FWHM values and PL peak positions for all investigated samples are presented in Table 4.

**Table 4:** Summary of Room Temperature Photoluminescence Characterization of CdS and Tb^3+^-CdS Q-Dot Glasses at Different Heat Treatment Temperatures Using a 450 nm Excitation Source, and Hydrogen Evolution Rate of CdS and Tb^3+^-CdS Q-Dot Glasses at Different Heat Treatment Temperatures Under Natural Sunlight

sample	heat treatment	Pl peak center (nm)	FWHM (nm)	lifetime (μs)	hydrogen evolution rate (μmol/h/0.5 g of catalyst)
2 wt % CdS only	without heat treatment	644	140 ± 3	1305 ± 4	0
	550 °C held for 6 h	668	130 ± 2	771 ± 8	3064
	575 °C held for 6 h	679	134 ± 3	643 ± 2	2965
	600 °C held for 6 h	690	134 ± 1	622 ± 3	2567
0.02 wt % Tb in 2 wt % CdS	without heat treatment	618	132 ± 4	1167 ± 2	0
	550 °C held for 6 h	648	156 ± 1	821 ± 4	3867
	575 °C held for 6 h	664	135 ± 2	662 ± 5	3748
	600 °C held for 6 h	695	123 ± 7	615 ± 1	3114
0.04 wt % Tb in 2 wt % CdS	without heat treatment	635	130 ± 2	806 ± 7	0
	550 °C held for 6 h	645	129 ± 2	645 ± 2	3667
	575 °C held for 6 h	676	128 ± 3	560 ± 6	3136
	600 °C held for 6 h	685	125 ± 2	532 ± 4	2629

Note that the PL lifetimes of the chemically dispersed Q-dots in a borosilicate glass provide insight into the efficiency and stability of photogenerated electrons in the excited state, as discussed by Hong et al. and Kommula et al.^57,58^ A short lifetime may lead to reducing photocatalytic activity, as the photogenerated electron–hole pairs may have less time to participate in contributing to the photodissociation of H_2_S via the H^+^ + HS^–^ reaction. Figure S2 illustrates the PL decay curves measured under 450 nm excitation for 2 wt % CdS and 0.04 wt % Tb^3+^-CdS-doped borosilicate glasses heat-treated at various temperatures. The PL decay curves were fitted with a double exponential function, and the average lifetime was obtained using eq 4.^59^
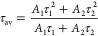
4where τ_1_ and τ_2_ represent the time constants for each exponential decay term and *A*_1_, and *A*_2_ represent their respective pre-exponential factors.

In this investigation, the analysis of data revealed the average lifetimes of 1305, 771, 643, and 622 μs for as-prepared and heat-treated 2 wt % CdS Q-dots in borosilicate glasses at 550, 575, and 600 °C. The decrease in the average lifetime is attributed to an increase in CdS Q-dot size and thus closer CdS–CdS Q-dot ion–ion interaction, which can result in faster recombination of electron–hole pairs.^58^ Moreover, the addition of 0.02 and 0.04 wt % Tb^3+^ ions to CdS Q-dots in borosilicate glass also led to a further decrease in the average lifetime with increasing heat treatment temperature as seen in Table 4. This can be attributed to Tb^3+^ ion concentration quenching, increased Q-dot size in borosilicate glass due to heat treatment temperature, and efficient energy transfer (ET) processes from the CdS Q-dot to the Tb^4+^ ion via the ET1 route, followed by the Tb^3+^ ion through route ET2, as illustrated in Figure 8A. The decrease in the CdS trap state lifetime may be attributed to the broad and strong absorption photon energy band of the Tb^4+^ ion, which could lead to efficient transfer of the absorbed energy to Tb^3+^ ions.

Furthermore, the lifetimes obtained from the CdS and Tb^3+^-CdS-doped borosilicate glasses are quite long compared to CdS in colloidal solution which is typically <200 ns.^60,61^ Thus, such long lifetimes measured from these samples may be ascribed to the reduced nonradiative decay processes originating from electron–phonon coupling in the glass matrix.

### 3.6 Photocatalytic Studies

**Figure 9:**
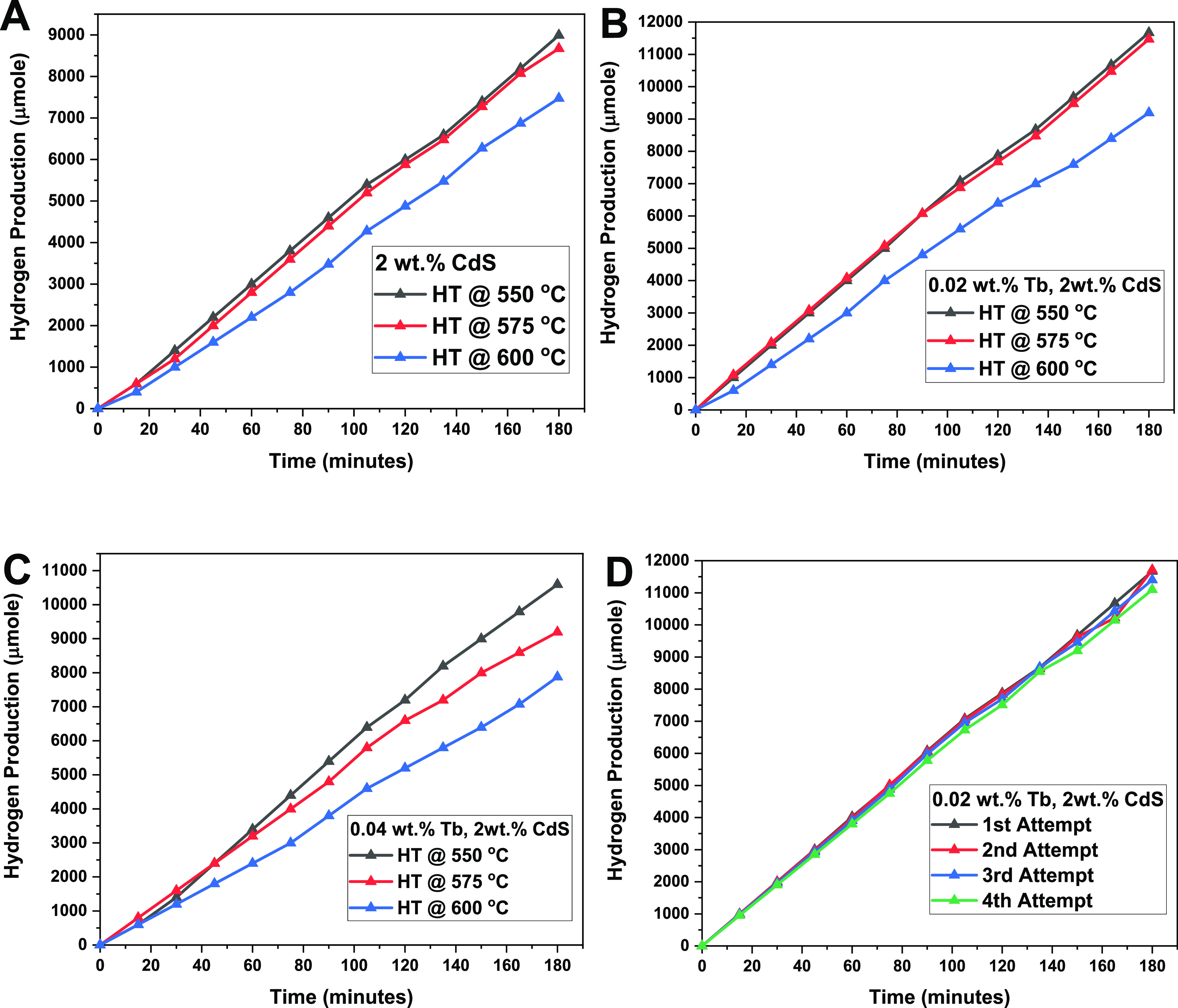
Time-dependant hydrogen production using (A) 2 wt % CdS, (B) 0.02 wt % Tb-doped CdS, (C) 0.04 wt % Tb-doped CdS, and (D) stability study of the sample 0.02 wt % Tb-doped CdS in glass treated at 550 °C.

The photocatalytic splitting of H_2_S was studied under natural sunlight using crushed powder of 2 wt % CdS and 0.02 and 0.04 wt % Tb^3+^-CdS quantum dot glasses as photocatalysts. The results of the photocatalytic activity of 2 wt % CdS and 0.02 and 0.04 wt % Tb^3+^-CdS quantum dot glass powders heat-treated between 550 and 600 °C for the photodecomposition of H_2_S into H_2_ gas are presented in Figure 9 and Table 4. As-prepared glass samples showed no photocatalytic activity and hence were not included in our results. The lack of photodissociation in as-prepared glass samples may be explained due the absence of a sufficiently large number of Q-dot nuclei which may be active in the energy transfer leading to photodissociation.

Average hydrogen evolution rates of 3064, 2965, and 2567 μmol h^–1^ were obtained for 2 wt % CdS Q-dot glass samples heat-treated at 550, 575, and 600 °C, respectively. It was observed that the photocatalytic activity of 2 wt % CdS Q-dot glass powder decreases as heat treatment temperature increases from 550 to 600 °C due to the increase in Q-dots size, as shown in Figure 9A. The hydrogen evolution rate of 2 wt % CdS Q-dot glass sample heat-treated at 550 °C is slightly higher than the hydrogen evolution rate reported by Apte et al.,^31^ where the authors synthesized CdS nanocrystalline of size 5–7 nm to generate hydrogen at the rate of 2945 μmol h^–1^ from the photodissociation of H_2_S using a 450 W Xenon lamp source, as shown in Table 1. Comparably better hydrogen evolution rate trends were also obtained for the 0.02 wt % Tb^3+^- and 0.04 wt % Tb^3+^-doped CdS Q-dot glasses as the heat treatment temperatures increase as shown in Figure 9B,C.

In this study, the highest H_2_ evolution rate was measured to be 3867 μmol h^–1^, which was attained from the 0.02 wt % Tb^3+^-doped CdS which was heat-treated at 550 °C having a small Q-dot size (≈4.8 nm) and a long lifetime (662 μs), as shown in Table 4. Doping CdS Q-dots with Tb^3+^ ions is more likely to be responsible for higher photocatalytic activity owing to a rise in energy transfer rate between the CdS Q-dot and Tb^3+^ ions. However, as the amount of Tb^3+^ ion increases, the effect of concentration quenching and nonradiative decay processes dominate, which decreases the lifetime and hydrogen evolution rate slightly. Thus, the photocatalytic activity of the 0.04 wt % Tb^3+^-CdS Q-dot glass heat-treated at various temperatures rendered a reduction in hydrogen evolution in comparison to the 0.02 wt % Tb^3+^-CdS Q-dot glass samples.

The 0.5 M KOH solution has a 12.5 pH (p*K*_a_ = 7.0), whereas, H_2_S is a weak diprotic acid (p*K*_a_ = 11.96). KOH dissolves in water and produces an OH^–^ anion. When H_2_S is bubbled through the 0.5 M KOH, it dissociates and maintains an equilibrium with HS^–^ ions as shown in the reaction in eq 6. Moreover, in nature, H_2_S absorbs ultraviolet light from the sunlight resulting in H_2_S dissociating into H^+^ and HS^–^ ions as illustrated in Figure 8A via route ET5.^62^

The CdS and Tb^3+^-doped CdS Q-dots absorb incident sunlight and generate photoexcited electrons (e^–^) and holes (h^+^). During the photocatalytic process, there are energy transfer mechanisms involving CdS and Tb^4+^/Tb^3+^ ions, facilitated by the ET1 and ET2 routes, respectively, as mentioned above. These processes act as recombination centers, effectively promoting the separation of electron–hole pairs. Due to the small size and large surface area of the Q-dots, the generated (e^–^) and (h^+^) were efficiently transported to the surface of the catalyst, making them readily available for photocatalytic activity. Through the ET4 pathway in Figure 8A, there is a resonance energy transfer from HS^–^ ions to the ground state of CdS or Tb^3+^-CdS quantum dots. This transfer contributes to the formation of H^+^ ions and S_2_ species, as shown in the HS^–^ ions relaxation via the reactions shown in eqs 8 and 9, respectively. Furthermore, it is worth mentioning that part of the ultraviolet–visible light photons, while in the excited state of H_2_S, can be transferred to the excited states of CdS or Tb^3+^-CdS quantum dots, as shown in ET3. This transfer generates additional electrons in the conduction band, which can subsequently react with the H^+^ ions to produce molecular hydrogen gas (H_2_) through the reaction in eq 10.

The reactions involved in the photocatalytic H_2_ generation via H_2_S splitting using CdS and Tb^3+^-CdS Q-dots are illustrated in eqs 5–10
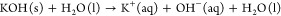
5
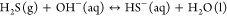
6
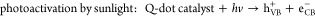
7
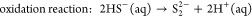
8
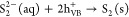
9
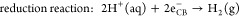
10The hydrogen production rate for Tb^3+^-doped CdS in glass is higher compared to that in CdS-only Q-dot glass. As a result of its exceptional photocatalytic activity, the stability of the 0.02 wt % Tb^3+^-doped CdS Q-dot glass heat-treated at 550 °C was assessed by repeating its photocatalytic hydrogen production under full natural solar light (Figure 9D) for 4 cycles. The remarkable stability of this sample was evidenced by the absence of a significant decrease in photocatalytic hydrogen production up to the 4th cycle, despite all four cycles being carried out under identical thermodynamic conditions. Our findings affirm that the doping of CdS and Tb^3+^-CdS Q-dots in a borosilicate glass matrix effectively shields the Q-dots from photocorrosion and sustains their photocatalytic properties, as opposed to bare CdS nanoparticles.

## 4 Conclusions

In conclusion, this study has demonstrated the potential of Tb^3+^-doped CdS Q-dots doped in a borosilicate glass matrix as a promising photocatalyst for the photodissociation of hydrogen sulfide (H_2_S) into hydrogen (H_2_) and elemental sulfur (S). The CdS and Tb^3+^-CdS Q-dot-doped borosilicate glasses were fabricated using a melt-quench method and were heat-treated between 550 and 600 °C for 6 h to control Q-dot nucleation and growth in the glass matrix. Detailed optical and microscopic analysis (UV–vis spectroscopy and TEM) confirms that the average Q-dot particle size is in the range of 3–6 nm, which is also supported by UV–vis transmittance and Brus model data. XRD confirmed the formation of hexagonal CdS and cubic Tb_2_O_3_ Q-dot structures in the borosilicate glass matrix. Besides, Raman spectroscopy showed the 1LO and 2LO longitudinal optical phonon modes of CdS. UV–vis transmittance spectra obtained from the CdS and Tb^3+^-CdS Q-dot glasses have displayed a red shift in cutoff wavelength as the Q-dot size increases with heat treatment. The optical band gap of the Q-dot glass samples decreases from 3.31 to 2.44 eV with increasing Q-dots crystal size indicating quantum confinement. Moreover, the PL emission exhibits a red shift toward NIR as the Q-dot size increases. The lifetimes of CdS and Tb^3+^-doped CdS Q-dot glasses are in the range of 1305–532 μs. These lifetimes decreased with increasing Q-dot size and increasing Tb^3+^ ion concentration. We have analyzed the dependence of Q-dot size and the heat treatment temperature on hydrogen evolution rates from H_2_S(g) under natural sunlight. The hydrogen evolution rate increased by 26.2% from 0.02 wt % Tb^3+^-doped CdS (3867 μmol/h/0.5 g) heat-treated at 550 °C when compared to CdS Q-dot glass with a similar heat treatment temperature (3064 μmol/h/0.5 g). This enhanced rate of H_2_ evolution was attributed to the stabilization of Q-dots and a long lifetime of 821 μs compared to other samples in this study. The photocatalytic properties of 0.02 wt % Tb^3+^-CdS sample heat-treated at 550 °C was observed to be preserved after being used for 4 cycles of hydrogen evolution.

## Supporting Information Available

Particle size distribution for Tb^3+^-CdS powder and photoluminescence decay curves of CdS and Tb^3+^-CdS Q-dots embedded in borosilicate glasses (PDF)
