# Supplementary material for: Engineering of Solar Energy Harvesting Tb3+-Ion-Doped CdS Quantum Dot Glasses for Photodissociation of Hydrogen Sulfide
Source: ACS Appl Energy Mater. 2023 Aug 29;6(17):8875–88. doi: 10.1021/acsaem.3c01488 (PMC10498422; doi:10.1021/acsaem.3c01488)
Supplement: Supplementary file 1 — ae3c01488_si_001.pdf [file ae3c01488_si_001.pdf]

Supplementary Information

Engineering of Solar-Energy Harvesting Tb^{3+}-ion doped CdS Quantum Dot Glasses for Photodissociation of Hydrogen Sulphide.

Mohanad Al-Murish, [a] Vijay Autade, [b] Eric Kumi-Barimah, [a] Rajendra Panmand, [b] Bharat Kale,[b*] and Animesh Jha[a*]

[a] School of Chemical and Process Engineering, University of Leeds, Leeds, LS2 9JT, UK

[b] Centre for Materials for Electronics Technology (C-MET), Ministry of Electronics and Information Technology (MeitY), Off Pashan Road, Panchawati, Pune,411008, India

* Corresponding authors. E-mail addresses: a.jha@leeds.ac.uk and bbkale@cmet.gov.in.

The particle size distributions of both samples, 0.02 wt.% Tb^{3+}-CdS powder heat treated at 550°C and 0.04 wt.% Tb^{3+}-CdS powder heat treated at 575°C for 6 hours, was analysed and presented in Figure S1. The results reveal that both samples exhibit a similar mean particle size of 28.6 and 29.6 micrometres, respectively. This similarity in particle size distribution indicates the consistency of the pulverization technique used on the samples.

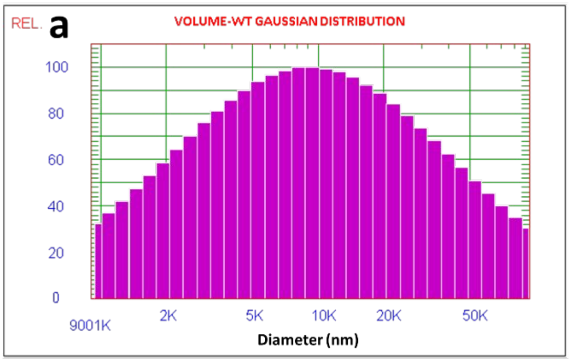

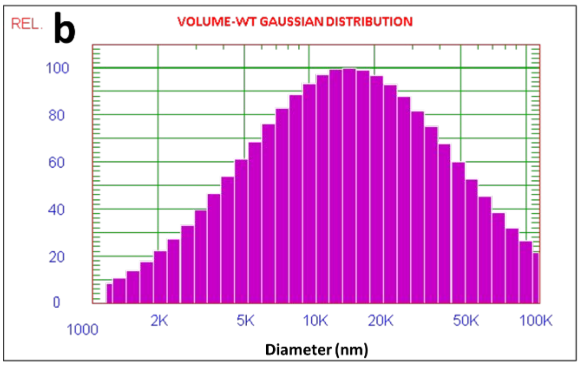

Figure S 1: Particle size distribution for (a) 0.02 wt. % Tb^{3+}-CdS powder heat treated at 550 °C and (b) 0.04 wt. % Tb^{3+}-CdS powder heat treated at 575 °C for 6 hours.

Photoluminescence decay curves presented in Figure S2A correspond to the quantum dot glasses containing 2 wt.% CdS. The figure shows the Pl decay curves for the as prepared sample and samples subjected to heat treatment at temperatures of 550°C, 575°C, and 600°C for 6 hours. Similarly, Figure S2B shows the Pl decay curves for the 0.04 wt.% Tb, 2 wt.% CdS as prepared sample and samples subjected to heat treatment at temperatures of 550°C, 575°C, and 600°C.

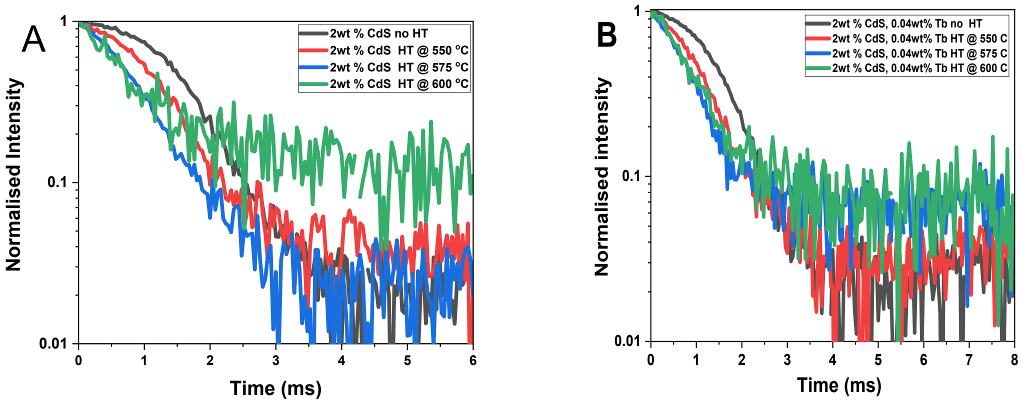

Figure S 2: Photoluminescence decay curves of Q-dots embedded in borosilicate glasses (A) 2 wt.%-CdS and (B) 0.04 wt. % Tb^{3+}-CdS at different heat treatment temperatures.
